# From malaria elimination to post‐elimination: a 10-year surveillance data study in Shanghai

**DOI:** 10.1186/s12936-021-03691-5

**Published:** 2021-04-26

**Authors:** Simin Dai, Min Zhu, Huanyu Wu, Yaoguang Zhang, Zhenyu Wang, Chengang Zhang, Xiaojiang Ma, Li Jiang

**Affiliations:** Shanghai Municipal Center for Disease Control & Prevention, Shanghai, People’s Republic of China

**Keywords:** Malaria, Surveillance, Shanghai, Elimination, Post-elimination

## Abstract

**Background:**

The aim of this study was to investigate and analyse the characteristics of malaria in Shanghai from 2010 to 2019 and to provide suggestions for areas with a similar elimination process in China in order to prompt development of strategies and interventions in the post-elimination stage.

**Methods:**

This was a cross-sectional study exploring the malaria characteristics during 2010–2019 in Shanghai, China. Malaria data from the Infectious Diseases Information Reporting Management System (IDIRMS) between 2010 and 2012 and data from the Parasitic Diseases Information Reporting Management System (PDIRMS) between 2013 and 2019 were combined for analysis in this study.

**Results:**

From 2010 to 2019, a total of 436 malaria cases were reported in Shanghai. Among them, 415 (95.18%) were imported from abroad, 19 (4.36%) were domestically acquired from other provinces, 1 (0.23%) case was caused by blood transfusion, and 1 (0.23%) had a long incubation. Only *Plasmodium vivax* was found in domestically indigenous cases; *Plasmodium falciparum* accounted for the largest proportion of imported cases. Domestically acquired cases were only reported in 2010–2011 and 88% occurred in June to September; no significant seasonal difference was observed for imported cases over the 10 years. No local transmission has occurred in Shanghai since 2012. The median interval from fever onset to diagnosis was 3 days. Between 2010 and 2019, among 308 foci, 33 were classified as potential transmission and dispersed in suburb areas (Minhang, Baoshan, Jiading, Pudong, Jinshan, Songjiang, Qingpu, Fengxian, and Chongming). Only *Anopheles sinensis* was present and the proportion of *Anopheles sinensis* in different species of mosquitoes under surveillance in Shanghai decreased from 2011 to 2019.

**Conclusions:**

Shanghai faces the challenge of malaria re-establishment caused by imported malaria in the post-elimination stage. Therefore, risk investigation and assessment should be carried out, and receptivity and susceptibility should be assessed for every point of focus. Training should be continued to strengthen facility staff capability, and multisectoral coordination and cooperation need to be conducted efficiently to maintain malaria elimination in Shanghai.

## Background

Malaria is a life-threatening public health issue that has aroused global concern for a long time. The disease is caused by one or more of five species of *Plasmodium* and female *Anopheles* mosquito serves as the transmission vector [1]. According to the 2020 World Malaria Report of the World Health Organization (WHO), about 229 million confirmed cases were reported in 2019, with approximately 409,000 deaths [2].

In 2010, China initiated the National Malaria Elimination Action Plan and no indigenous cases have been reported in the country since 2017 [3]. The city of Shanghai is situated on the banks of the Yangtze River Delta in East China, which was once a highly endemic area for malaria; in the early 1960s, *Plasmodium vivax* predominated and the incidence peaked at 3000 per 100,000 people. After substantial support and efforts made from various stakeholders, Shanghai city hosted the national malaria elimination pilot project and first launched its malaria elimination programme in 2009 [4]. No indigenous cases have been reported in the city from 2010 through to 2019. Efficient efforts made mainly involved strictly implementing a local “1−2−3+1” strategy for malaria case management (which refers to reporting of malaria cases within 1 day, case confirmation and investigation within 2 days, foci investigation and response to prevent further transmission within 3 days, and continuous case follow-up to prevent re-establishment for 1 month), as well as promoting multisectoral coordination and cooperation mechanism.

Despite the remarkable achievements in malaria elimination made in Shanghai, rapid development and globalization has seriously increased population mobility domestically and internationally, which render imported malaria a great challenge in Shanghai. This study investigated and analysed the characteristics of malaria in Shanghai from 2010 to 2019. Because other areas in China that are working toward elimination face the same challenge as Shanghai, results from this study could have practical implications and provide inspiration for other regions of the country to develop post-elimination strategies and interventions in a timely manner.

## Methods

### Study site

The study was conducted in Shanghai city, one of the four municipalities directly under the central government, located in eastern China. The city borders Jiangsu Province as well as Zhejiang and Anhui provinces, both historically severe malaria endemic areas. It is worth mentioning that Anhui has had three malaria endemic periods in its history, with an average disease incidence rate of 22.76%. Although Anhui qualified for national malaria elimination assessment in 2019, many of its residents choose to move to Shanghai, where they become part of the city’s floating population [5]. Shanghai has 16 districts (7 urban areas, 9 rural areas), with total area of 6340.50 km^2^. According to a government report in 2019, Shanghai has about 24.23 million inhabitants, and 40% of them are migrants, who came from other provinces in China. As a mega-city that combines finance, trade, shipping, and technological innovation, Shanghai has long attracted worldwide attention. Over the span of the past 10 years, the population has grown to approximately 9 million migrant citizens each year. Shanghai’s trading partners have also expanded from more than 20 countries in the early 1970s to more than 200 countries and regions today.

### Study design

This research conducted a cross-sectional study to explore the malaria characteristics during 2010–2019 in Shanghai. Most of the cases reviewed and analysed were gathered from the Infectious Diseases Information Reporting Management System (IDIRMS), which covers all hospitals at the municipal and county levels. In the second half of 2012, the government of China established the Parasitic Diseases Information Reporting Management System (PDIRMS), to determine whether every malaria case was indigenous or imported. To some degree, the PDIRMS contains more comprehensive and detailed information than the IDIRMS, which was set up in 2004 after the outbreak of severe acute respiratory syndrome (SARS). Considering the data quality, herein, malaria data from the IDIRMS between 2010 and 2012 and data from the PDIRMS between 2013 and 2019 were combined for the analysis in this study. The data for the study, based upon annual case distribution, were selected using reporting data and reporting area whereas data for the analysis on foci evolution at different stages were selected using case residence data. All data from Hong Kong, Macao, and Taiwan were excluded from the statistical analyses [6].

In this work, the 10-year study period was divided into three parts for further discussion; 2010–2012 is the initial stage of malaria elimination, in which the PDIRMS was first established during the second half of 2012 and domestically cases still existed in Shanghai. In the formal malaria elimination phase (2013–2016), the Shanghai government initiated various malaria elimination tasks in an orderly manner so as to precisely identify the source of each case. The years between 2017 and 2019 represent the post-elimination phase, during which finding and treating every imported case in time was the priority.

### Characteristics of included cases

During the span of this 10-year, 436 malaria cases were reported by 70 hospitals and all were included in this study. Clinically diagnosed cases were defined as patients with malaria-related symptoms but with negative results on blood examination, and laboratory-confirmed cases were positive in microscopy examination, polymerase chain reaction (PCR), or rapid diagnostic tests. Indigenous case refers to malaria acquired by mosquito transmission in an area within China. Any cases defined as imported malaria referred to patients who acquired the illness from a known malaria-prevalent region outside of China. All imported cases were included in this study.

### Focus classification

According to the Technical Programme to Eliminate Malaria in Shanghai, a natural village is considered the smallest unit of focus. “Focus with transmission” refers to a focus with indigenous or imported secondary cases. “Focus with possible transmission” refers to a focus with both imported cases and ecological factors necessary for malaria transmission. “Focus with no transmission” refers to a focus with imported cases but without vectors, or a focus with imported cases and vectors but not being within the transmission season.

### Statistical analysis

A descriptive analysis was performed using SAS Version 9.4 (SAS Institute, Cary, NC, USA). The χ^2^ test was used to compare the seasonal difference in temporal distribution of malaria cases, and to explore the evolution of foci with potential transmission in different periods. A *P* value of < 0.05 was considered statistically significant. The seasonal index was used to understand the seasonal patterns of malaria incidence. The index was calculated as the case number for a given month (i.e., January) divided by the mean number of cases in that corresponding month during 2010–2019. No obvious seasonal pattern was expected if the seasonal index in each month was close to 1.0.

## Results

### Epidemiological study in Shanghai City, 2010–2019

From 2010 to 2019, a total of 436 malaria cases were reported, and all were laboratory-confirmed. Among them, 415 (95.18%) were imported whereas 19 (4.36%) were “domestically imported” from other provinces; 1 case (0.23%) was caused by blood transfusion and 1 case (0.23%) had a long incubation with *Plasmodium malariae*. All domestic cases were identified as *Plasmodium vivax*. Among the imported cases, 4 *Plasmodium* species were identified: *P. vivax* (n = 44, 10.60%), *P. falciparum* (n = 306, 73.73%), *Plasmodium ovale* (n = 37, 9.92%), and *P. malariae* (n = 16, 3.86 %), as well as 12 undifferentiated cases (n = 12, 2.89%). In addition, 4 deaths caused by *P. falciparum* were reported, one each in 2011, 2014, 2015, and 2019 (Table 1).

**Table 1:** Malaria in Shanghai city, 2010–2019

Year	No. cases	*Plasmodium* species					Case classification				No. of deaths
		*P. falciparum*	*P. vivax*	*P. ovale*	*P. malariae*	Undifferentiated#	Imported	Domestically imported	Long incubation	Blood infection	
2010	45	12	21	0	0	12	31	14	0	0	0
2011	49	39	8	0	2	0	44	5	0	0	1
2012	26	19	6	1	0	0	26	0	0	0	0
2013	45	35	5	3	2	0	44	0	0	1	0
2014	46	30	6	7	3	0	45	0	1	0	1
2015	42	32	6	3	1	0	42	0	0	0	1
2016	46	33	6	6	1	0	46	0	0	0	0
2017	60	48	2	6	4	0	60	0	0	0	0
2018	40	30	3	4	3	0	40	0	0	0	0
2019	37	28	0	7	2	0	37	0	0	0	1
Total	436	306	63	37	18	12	415	19	1	1	4

# In 2010, no *Plasmodium* species were clearly identified in 12 cases

**Fig. 1:**
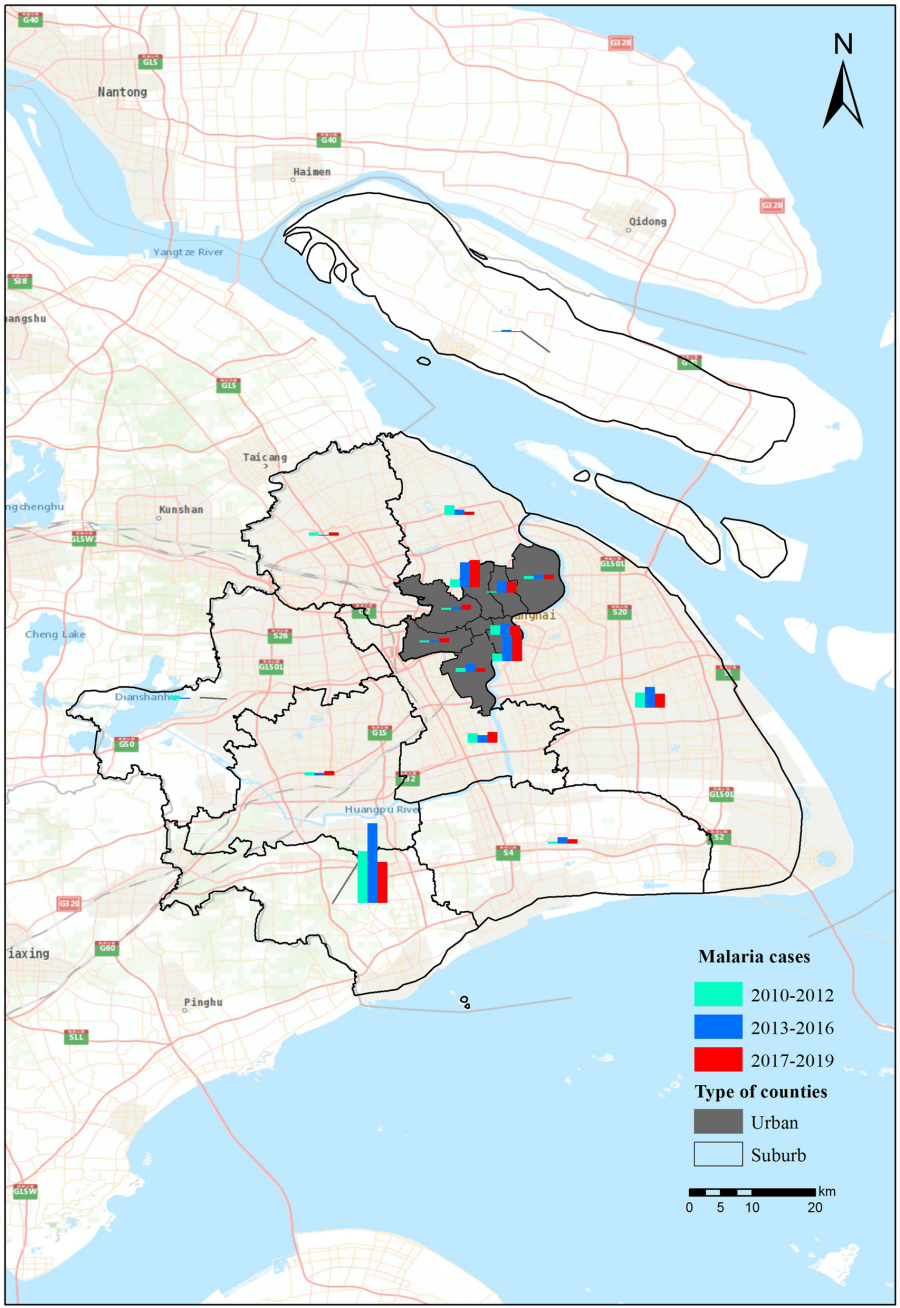
Regional distribution of reported malaria cases in Shanghai, 2010–2019

Malaria cases were reported in all 16 districts of Shanghai during 2010–2019, and most (n = 160, 36.70%) were reported in Jinshan district, followed by Jing’an district (n = 55, 12.61%) and Pudong district (n = 4 6, 10.55%). Between 2010 and 2012, 120 cases were reported, with 40% from Jinshan and 11.67% from Pudong. During 2013–2016 and 2017–2019 a total of 179 and 137 cases were reported, respectively, with the top three reporting areas in these periods being Jinshan (41.34%, 12.84%), Jing’an (27.74%, 18.25%), and Pudong (10.61%, 9.49%), respectively (Fig. 1). Malaria cases comprised 89.91% in men and 10.09% in women. The median age of reported malaria cases was 38 (range 1–76) years and 57.34% of cases occurred in individuals aged 30–49 years.

A total of 36 countries in Africa were reported as the sources of imported cases. Nigeria (n = 65, 17.33%), Angola (n = 25, 6.67%), and Democratic Republic of the Congo (n = 25, 6.67%) were the main source countries. Cases imported from Africa were predominantly infected with *P. falciparum* (n = 301, 80.27%). Countries in Southeast Asia, including Indonesia (n = 11, 64.71%) and Myanmar (n = 5, 29.41%) were considered another major source. These cases were mostly infected with *P. vivax* (n = 15, 88.24%). One case with *P. falciparum* infection from Haiti and two cases infected with *P. vivax* from Papua New Guinea were also reported. Domestic cases imported from other provinces were only reported during 2010–2011; source provinces included Anhui (n = 15, 78.95%), Henan (n = 2, 10.53 %), Yunnan (n = 1, 5.26%), and Jiangsu (n = 1, 5.26%) (Table 2).

**Table 2:** Distribution of imported cases by country of acquisition and Plasmodium species in Shanghai, 2010–2011

Continent	Country	*Plasmodium* species					Total
		*P. falciparum*	*P. vivax*	*P. ovale*	*P. malariae*	Undifferentiated	
Asia	India	1	13	0	1	1	16
	Indonesia	2	10	0	0	0	12
	Myanmar	0	5	0	0	2	7
	Pakistan	1	0	0	0	0	1
	Laos	0	1	0	0	0	1
Africa	Nigeria	65	1	4	2	3	75
	Angola	25	2	3	3	1	34
	Democratic Republic of the Congo	25	1	4	0	0	30
	Ghana	23	0	3	1	0	27
	Cameroon	22	0	4	2	0	28
	Gabon	15	0	4	2	0	21
	Equatorial Guinea	16	1	2	0	1	20
	Republic of Guinea	15	0	2	0	0	17
	Côte d’Ivoire	10	0	0	0	2	12
	Other countries (27)	85	8	11	5	2	111
Latin America	Haiti	1	0	0	0	0	1
Oceania	Papua New Guinea	0	2	0	0	0	2
Total		306	44	37	16	12	415

**Fig. 2:**
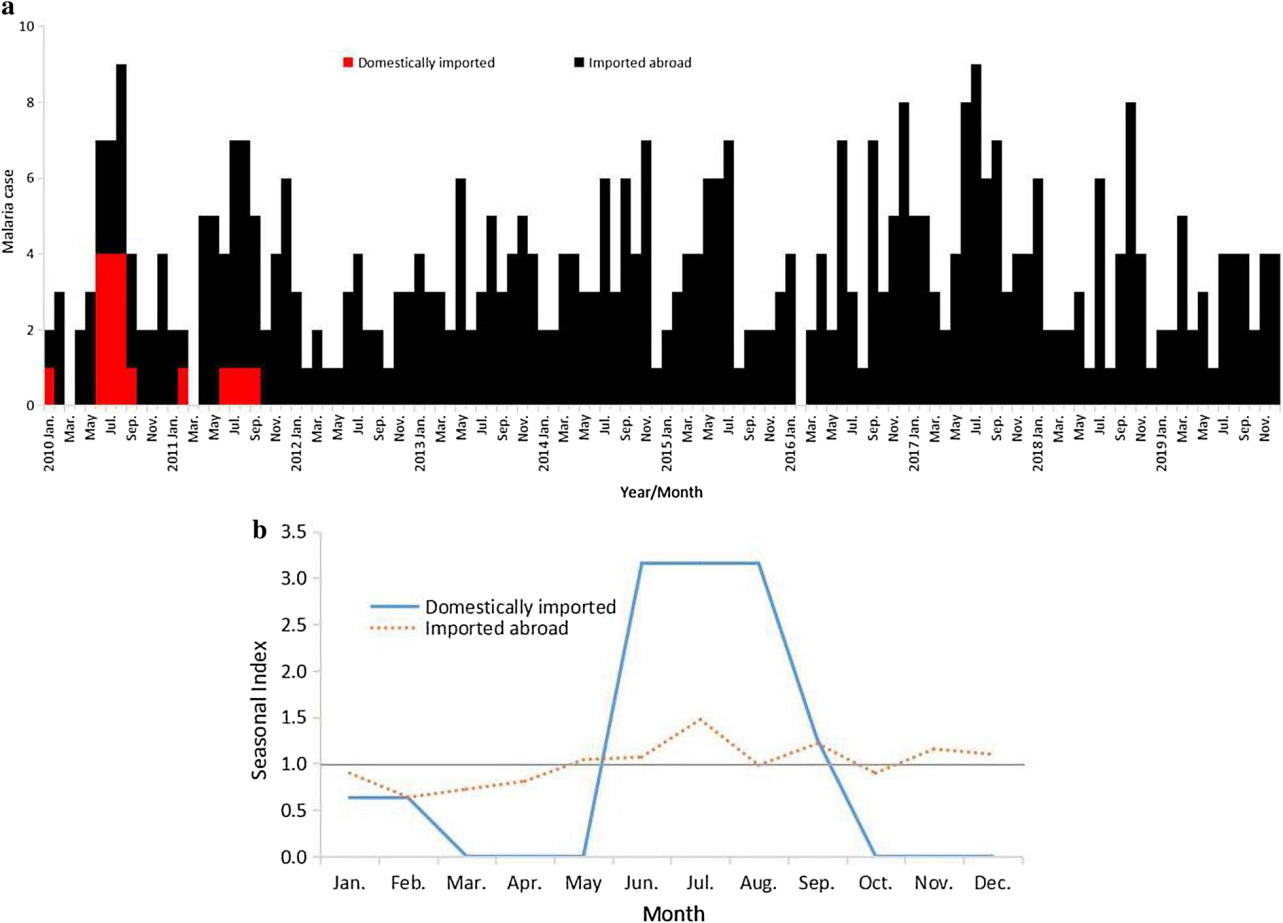
Temporal distribution of malaria cases in Shanghai, 2010–2019

The temporal distribution of malaria cases varied. As for domestically imported cases, most malaria cases during 2010–2011 occurred yearly from June to September (n = 16, 88.89%); no significant seasonal difference was observed for imported cases (χ = 0.536, P > 0.05). Since 2012, Shanghai only identified imported cases, which were reported in nearly every month during the time span of 10 years (Fig. 2).

### Interval from onset of fever to diagnosis

**Fig. 3:**
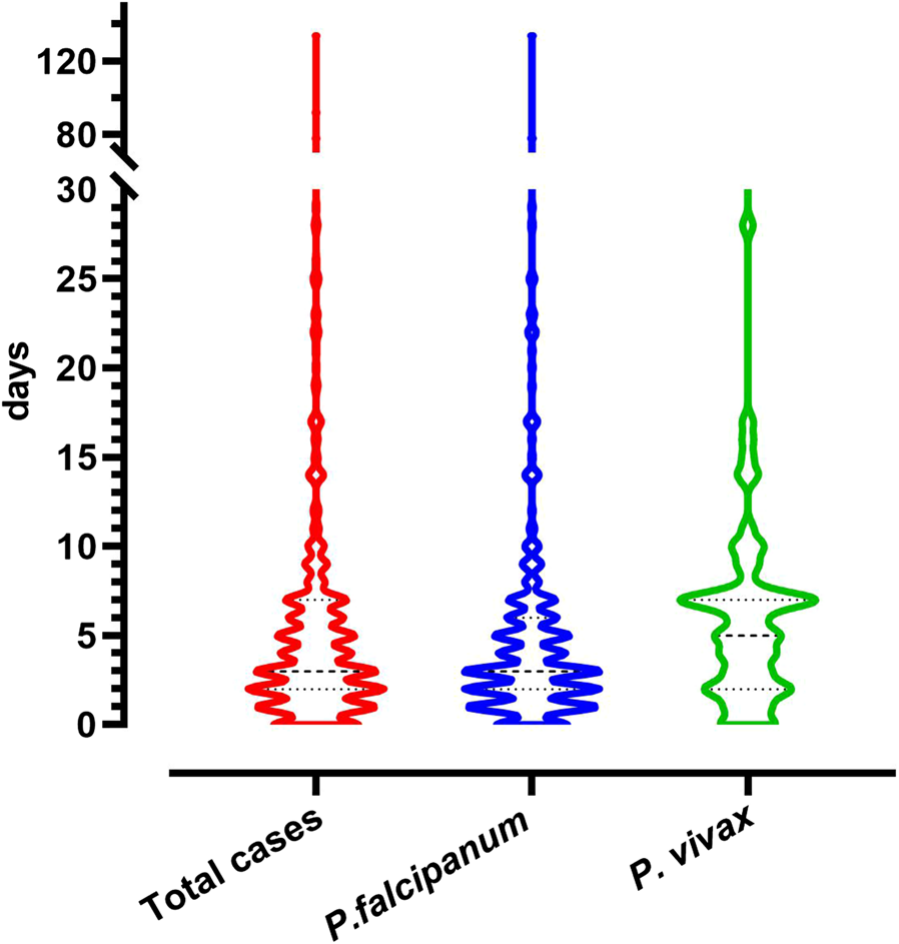
Time interval from fever onset to diagnosis of malaria in Shanghai, 2010–2019

The number of days elapsed from the onset of fever to diagnosis was recorded. The results demonstrated that the median interval from fever onset to diagnosis was 3 days (interquartile range [IQR], 2–7 days). As for *P. vivax*, the median interval was 5 days (IQR, 2–7 days), with the shortest period of 0 days and the longest period of 38 days. *P. falciparum* had a 3-day median interval (IQR, 2–6 days) (Fig. 3).

### Foci identification and classification

**Fig. 4:**
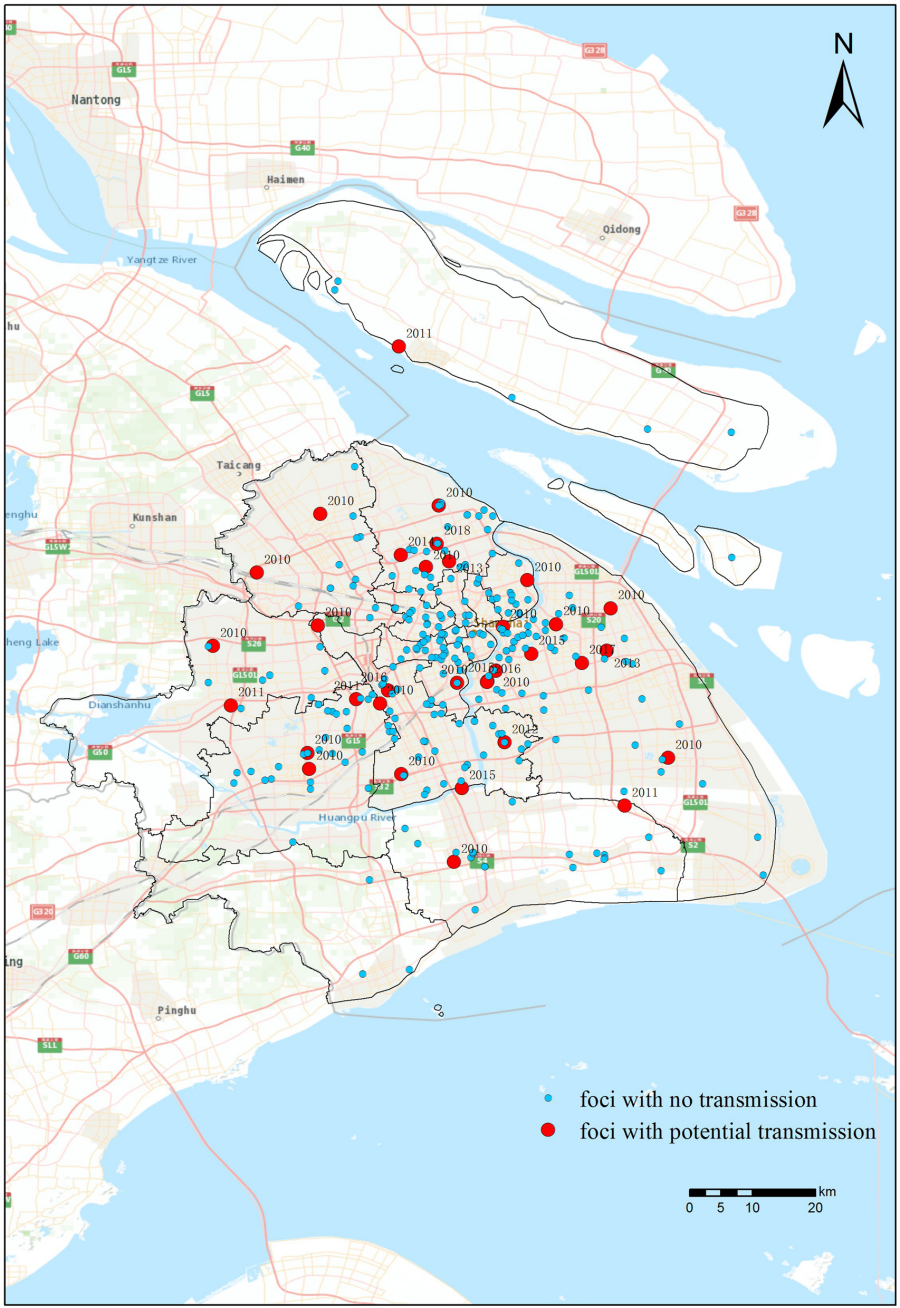
Foci classification for malaria cases in Shanghai, 2010–2019

Among 436 cases reported between 2010 and 2019, a total of 308 foci were used for foci identification and classification. The 308 foci were classified as 33 foci with potential transmission and 275 foci with no transmission. All 33 foci with potential transmission were dispersed in suburb areas (n = 33, 100%) and 22 of them were reported between 2010 and 2011. The 275 foci with no transmission were mostly in urban districts (n = 78, 28.36 %) (Fig. 4). During the malaria elimination stage (2010–2016), 14.01% (31/220) of foci were classified as potential transmission, all of which were distributed in rural areas; this number dramatically decreased to 2.27% (2/88) in the post-malaria elimination stage (2017–2019) (χ = 9.177, P < 0.05).

### Vector surveillance

Vector surveillance conducted from 2010 to 2019 showed that only *Anopheles sinensis* was present in Shanghai. According to yearly reports on mosquito surveillance from Shanghai, a total of 2861 *An. sinensis* mosquitoes were captured using the carbon dioxide lamp trapping method during 2010–2019, accounting for 2.36% of the total number of mosquitoes, (e.g. *Culex. pipiens pallens*, *Culex tritaeniorhynchus*, *Aedes albopictus*), with a range from 0.86 to − 6.90% within the 10 years. During this period, the proportion of *Anopheles sinensis* in different species of mosquitoes under surveillance in Shanghai of *An. sinensis* decreased from 6.90% to 2011 to 1.36% in 2019. Most *An. sinensis* mosquitoes were distributed in suburban areas of Shanghai, including Chongming (n = 599, 20.94%), Jiading (n = 583, 20.38%), and Songjiang (n = 479, 16.74%).

## Discussion

Over the past few decades, the integrated control and elimination strategy and interventions have contributed to sharply declining incidence rate of indigenous malaria in China. Shanghai city, historically plagued by *P. vivax* malaria infections, has also achieved great success as the first city to meet the national and WHO assessment criteria for malaria elimination [6].

No local transmission has been reported in Shanghai city from 2010 to 2012, except 19 *P. vivax* cases imported from other provinces in China. During the formal malaria elimination phase (2013–2016) and post-elimination phase (2017–2019), only imported cases from outside China were reported. Among all imported cases, most came from Africa, with *P. falciparum* as the main species infecting humans [7]. Apart from that, after 2012, *P. ovale* and *P. malariae* infections have seen an increase, mainly imported from western and central Africa. During these three stages, Jinshan, Pudong and Jin’an districts saw relatively high reported case number. In the 10-year span of the present study, there was no significant difference in reported cases each year.

This study observed that in the span of 10 years examined here, no foci with transmission have been found. Between 2010 and 2011, the largest populations of *P. vivax* were reported between June and August. The time interval between onset of fever to diagnosis was investigated, which may reflect patients’ willingness to seek medical treatment or the intention to avoid receiving a malaria diagnosis. This study demonstrated that the median time interval from the onset of fever to diagnosis was 5 days for *P. vivax*, which exceeds the 3 days reported in some studies [8].

During this 10 years, Jinshan district always led other areas in case reporting numbers owing to the fact that the Public Health Clinical Center, the only infectious diseases hospital at municipal level, is located in Jinshan district. Pudong has an international airport, which largely explains its huge transnational population movement; Jin’an district possesses a large hospital with professionals who are skilled in the diagnosis of infectious disease and is assigned as the only facility in Shanghai able to store anti-malarials. The establishment of a reference laboratory, which uses PCR to reconfirm *Plasmodium* species helps to explain why *P. ovale* and *P. malariae* infections have seen an increase after 2012, since *P. vivax* and *P. ovale* can easily be confused when using only microscopy [9].

The post-elimination stage has seen a remarkable decrease in the proportion of foci with potential transmission; this may be partly attributed to a wide array of reasons, such as rapid improvement in living environments, the management and supervision of both the domestic and international migrant populations, continuous improvement in medical conditions, as well as the strengthening of mosquito prevention awareness. Nevertheless, compared with the monitoring of migrant populations, vector control could be more challenging as it is impossible to completely eliminate mosquito populations. June and August saw the largest populations of *P. vivax* between 2010 and 2011, and this may be for two reasons. First, much research has revealed that temperatures between 25 and 28 °C are preferred by *Anopheles* mosquitoes in China [10], which is consistent with the temperatures during the transmission season from June to August. Second, many workers in Shanghai return to their hometowns to perform agricultural work during this period, and those areas tend to be environments highly conducive to malaria transmission. Although no indigenous cases have been reported and the number of *P. vivax* has been decreasing in Shanghai since 2010, this study suggested that most patients are likely to live in suburbs where *Anopheles* mosquitoes can breed easily [11]. The results herein provide evidence that case investigation and foci response should be carried out more precisely between these periods to block potential transmission.

According to a previous paper, a longer time interval will increase the malaria transmission time when *P. vivax* appears earlier in infected patients [12]. In addition, a longer interval will increase risk for malaria re-establishment as *An. sinensis* is distributed throughout Shanghai. With regard to the 306 *P. falciparum* cases reported in Shanghai, the interval from fever onset to diagnosis was 3 days, which is longer than the national average of 2 days [13]. *Plasmodium falciparum* has a shorter incubation period and more rapid onset than *P. vivax*, with serious physiological damage; therefore, timely diagnosis and treatment are urgently needed.

In summary, Shanghai has had notable success in malaria elimination. Considering the comparatively low *Anopheles* mosquito density as well scattered *P. vivax* case distribution during 2017–2019, Shanghai has a low risk of malaria re-establishment. However, owing to the increase in imported case in Shanghai, training to physicians to ensure accurate and timely diagnosis as well as appropriate treatment of malaria cases, especially patients with *P. falciparum* infection, is strongly encouraged. At the same time, Shanghai should continue to widely implement and promote its local “1−2−3 + 1” strategy for malaria case management, tailored to the “1−3−7” strategy (case reporting within 1 day, case investigation within 3 days, and focus investigation and action within 7 days) launched in 2012 for carrying out case follow-up and conducting risk assessment reporting within 1 month, which is conducive to preventing re-establishment after foci investigation [14]. As an international metropolis with a large annual migrant population, an effective surveillance system in Shanghai still must be carefully planned and managed to ensure timely recognition and prompt response. In certain periods,such as the Chinese New Year holiday, health care staff should carry out active surveillance among all mobile labourers and other travellers after they return from abroad. Moreover, the knowledge required to prevent and treat different *Plasmodium* species should been improved among medical staff and health education information on malaria risks and protection should be provided to travellers before they travel abroad. Furthermore, multisectoral coordination and cooperation mechanisms should continue to be strengthened, especially when facing the groups from Africa on arrival [15].

## Data Availability

Data supporting the conclusions of this article are included within the article. The datasets used and/or analysed during the present study are available from the corresponding author upon reasonable request.

## Abbreviations

IDIRMS: Infectious Diseases Information Reporting Management System
PDIRMS: Parasitic Diseases Information Reporting Management System
WHO: World Health Organization
PCR: Polymerase chain reaction
